# Intravenous cangrelor use for neuroendovascular procedures: a two-center experience and updated systematic review

**DOI:** 10.3389/fneur.2023.1304599

**Published:** 2023-12-05

**Authors:** Harsh Desai, Mohammed Maan Al-Salihi, Rami Z. Morsi, Omar R. Vayani, Sachin A. Kothari, Sonam Thind, Julián Carrión-Penagos, Archit Baskaran, Ammar Tarabichi, Veronica A. Bonderski, James E. Siegler, Mary Hahn, Elisheva R. Coleman, James R. Brorson, Scott J. Mendelson, Ali Mansour, Guilherme Dabus, Michael Hurley, Shyam Prabhakaran, Italo Linfante, Tareq Kass-Hout

**Affiliations:** ^1^Department of Neurology, University of Chicago, Chicago, IL, United States; ^2^Department of Neurological Surgery, School of Medicine and Public Health, University of Wisconsin, Madison, WI, United States; ^3^Pritzker School of Medicine, University of Chicago, Chicago, IL, United States; ^4^Department of Neurology, DENT Neurologic Institute, Amherst, NY, United States; ^5^Department of Pharmacy, University of Chicago, Chicago, IL, United States; ^6^Department of Neurology, Stony Brook University Hospital, Stony Brook, NY, United States; ^7^Department of Neurology, Baptist Cardiac and Vascular Institute, Miami, FL, United States; ^8^Department of Radiology, University of Chicago, Chicago, IL, United States

**Keywords:** cangrelor, anti-platelet therapy, endovascular, systematic review, stroke

## Abstract

**Background:**

The optimal antiplatelet therapy regimen for certain neuroendovascular procedures remains unclear. This study investigates the safety and feasibility of intravenous dose-adjusted cangrelor in patients undergoing acute neuroendovascular interventions.

**Methods:**

We conducted a retrospective chart review of all consecutive patients on intravenous cangrelor for neuroendovascular procedures between September 1, 2020, and March 13, 2022. We also conducted an updated systematic review and meta-analysis using PubMed, Scopus, Web of Science, Embase and the Cochrane Library up to February 22, 2023.

**Results:**

In our cohort, a total of 76 patients were included [mean age (years): 57.2 ± 18.2, males: 39 (51.3), Black: 49 (64.5)]. Cangrelor was most used for embolization and intracranial stent placement (*n* = 24, 32%). Approximately 44% of our patients had a favorable outcome with a modified Rankin Scale (mRS) score of 0 to 2 at 90 days (*n* = 25/57); within 1 year, 8% of patients had recurrent or new strokes (*n* = 5/59), 6% had symptomatic intracranial hemorrhage [sICH] (4/64), 3% had major extracranial bleeding events (2/64), and 3% had a gastrointestinal bleed (2/64). In our meta-analysis, 11 studies with 298 patients were included. The pooled proportion of sICH and intraprocedural thromboembolic complication events were 0.07 [95% CI 0.04 to 1.13] and 0.08 [95% CI 0.05 to 0.15], respectively.

**Conclusion:**

Our study found that intravenous cangrelor appears to be safe and effective in neuroendovascular procedures, with low rates of bleeding and ischemic events. However, further research is needed to compare different dosing and titration protocols of cangrelor and other intravenous agents.

## 1 Introduction

The benefits of endovascular intervention for neurologic disease must be weighed against the risks, namely that of peri-procedural hemorrhage and cerebral infarction (1). Optimizing antithrombotic regimens during neuroendovascular procedures has become essential, particularly as new antiplatelet and anticoagulant agents are introduced to the market (2). Antiplatelet agents remain crucial during neuroendovascular interventions because the introduction of devices, such as stents, flow diverters, and embolization coils, promotes the adherence of fibrinogen and other plasma proteins to these foreign bodies, leading to thrombosis and obstruction (3). Commonly used antiplatelet agents include cyclo-oxygenase (COX)-1 inhibitors (e.g., aspirin), dipyridamole, glycoprotein IIb/IIIa inhibitors (e.g., tirofiban, eptifibatide), P2Y_12_ adenosine diphosphate (ADP) inhibitors (e.g., clopidogrel, prasugrel, ticagrelor), and phosphodiesterase (PDE) inhibitors (e.g., cilostazol) (2, 4, 5). However, these drugs come with the challenge of achieving a balance between thrombosis prevention and bleeding promotion (6).

Cangrelor is a newer generation P2Y_12_ ADP inhibitor that has demonstrated promise in achieving this balance. This drug has ideal pharmacokinetic properties, with rapid onset and short duration, allowing for more finer control of platelet inhibition (7). Cardiology trials have demonstrated that the drug effectively prevents periprocedural thrombotic events without significant increase in severe bleeding as well as a reduction in myocardial infarction and in-stent thrombosis (8, 9). Multiple case series and retrospective reviews have since demonstrated the therapeutic efficacy and safety of cangrelor in acute neuroendovascular interventions (10–16). Despite this, the evidence related to the safety and efficacy of intravenous cangrelor use for acute neuroendovascular interventions remains in question without any existing standardization in its utilization across institutions (17).

In this two-center study, we aimed to investigate the safety and feasibility of intravenous cangrelor in patients undergoing acute neurovascular interventions, such as acute stenting or embolization, and compare two different dosing titration regimens. We also aimed to conduct an updated systematic review and meta-analysis to assess the available literature about intravenous cangrelor in patients undergoing neuroendovascular procedures and pool the relevant data regarding cangrelor efficacy and safety.

## 2 Materials and methods

### 2.1 Retrospective study

#### 2.1.1 Patient population

This is a two center retrospective study of all consecutive patients who underwent neuroendovascular procedures and were placed on intravenous cangrelor between September 1, 2020, and March 13, 2022. The Institutional Review Board at the University of Chicago approved the study protocol, and the need for informed consent was waived.

We included patients in this study if they met the following inclusion criteria: (1) age ≥ 18 years; patients underwent neuroendovascular procedures, such as endovascular thrombectomy (EVT), intracranial or carotid stenting, or aneurysm embolization using various techniques (e.g., stent-assisted coiling, flow diverter placement, etc.); and use of intravenous cangrelor regardless of duration.

#### 2.1.2 Cangrelor protocol

Immediately after each neuroendovascular procedure, an intravenous (IV) bolus of 15 to 30 mcg/kg cangrelor was administered, followed by a starting maintenance infusion of 2 to 4 mcg/kg/min, depending on each institutional protocol (see Table 1). Due to the retrospective nature of the study the two protocols were only utilized at the respective centers where they were created. The cangrelor infusion was then titrated to goal P2Y_12_ reaction unit (PRU) level of 50 to 150 and maintained until bridging to oral antiplatelets was done. We used the VerifyNow P2Y_12_ (Accumetrics, San Diego, CA) to quantify the PRU level (18). P2Y_12_ reaction unit assays are typically done daily. Bridging to oral antiplatelet therapy is considered when PRU goal has been met, but can be provider-dependent, especially if there are other planned procedures (e.g., gastrostomy tube insertions, etc.). Titration to oral antiplatelet therapy (e.g., clopidogrel or ticagrelor) occurred before or immediately post-cangrelor infusion discontinuation, and patients were subsequently discharged on dual antiplatelet therapy (e.g., aspirin and clopidogrel, aspirin and ticagrelor). For example, patients were given a ticagrelor loading dose 2 h prior to cangrelor infusion cessation versus stopping the infusion and immediately giving a clopidogrel load. Table 1 further summarizes the two cangrelor infusion protocols and the timing of bridging with cangrelor infusion.

**Table 1:** Detailed outline of different cangrelor infusion protocols.

Protocol characteristics	Protocol	
	A	B
Loading dose of cangrelor (mcg/kg)	±15	30
Maintenance dose of cangrelor (mcg/kg/min)	2	4
Duration of cangrelor administration (median, hours)	N/A^a^	2
Starting dose of aspirin (mg)	325	325
Type and dose of antiplatelet bridging therapy (mg)	Ticagrelor 90 or clopidogrel (300 or 600)	Ticagrelor 180
Duration of bridging therapy (hours)	2 (if ticagrelor), 0 (if clopidogrel)	2
Type of antiplatelet therapy on discharge	Aspirin and (clopidogrel or ticagrelor)	Aspirin and ticagrelor

a Contingent on P2Y12 reaction units and the discretion of the neurointerventionist.

#### 2.1.3 Patient outcomes

For patients with acute internal carotid artery (ICA) or another intracranial vessel occlusion, we defined favorable functional outcome as a modified Rankin Scale (mRS) score of 0 to 2 at 90 days. We also assessed for the following outcomes at 6 months to 1 year: recurrent or new strokes; symptomatic intracranial hemorrhage (sICH), defined as any intracranial hemorrhage associated with worsening neurologic exam, clinical deterioration or death, adapted from the Heidelberg Bleeding Classification (19); asymptomatic intracranial hemorrhage (aICH), defined as any intracranial hemorrhage without worsening neurologic exam, clinical deterioration or death; in-stent thrombosis; gastrointestinal bleeding events; or retroperitoneal hematoma.

### 2.2 Statistical analysis

Baseline characteristics are summarized using median and interquartile range (IQR) or mean and standard deviation for continuous variables. Frequency distribution was used for categorical variables, such as NIHSS and mRS scores. Dichotomous outcome measures, such as mortality at discharge and at 90 days, favorable functional outcomes at discharge and at 90 days, sICH, aICH, major extracranial and gastrointestinal bleeding events, new or recurrent and disposition between the two protocol groups were analyzed using chi-squared or Fisher’s exact test when appropriate. All statistical analysis of the cohort study was completed using SPSS V28.0.1.1.

### 2.3 Systematic review

#### 2.3.1 Standardized reporting and registration

We designed our systematic review and meta-analysis according to the Cochrane Handbook for Systematic Reviews of Interventions and the Preferred Reporting Items for Systematic Reviews and Meta-analyses (PRISMA) guidelines (20, 21). We registered our systematic review protocol on PROSPERO (CRD42023403598).

#### 2.3.2 Data sources and searches

With the help of an information specialist, we searched the following databases from inception to February 22, 2023: PubMed, Scopus, Web of Science, Embase and the Cochrane Library. We did not use any language restrictions. We used the following keywords: cangrelor, AR-C69931MX, Kengreal®, Canreal, stroke, strokes, CVA, brain vascular accident, apoplexy, cerebrovascular, and neuroendovascular. We also hand-searched the grey literature and reference lists of included studies to decrease the risk of publication bias.

#### 2.3.3 Study selection and data extraction

We included studies assessing the safety and efficacy of intravenous cangrelor in the setting of acute neuroendovascular procedures, such as with stroke or aneurysm treatment. Editorials, commentaries, literature and systematic reviews, and case reports were excluded from the systematic review. *In vitro*, animal and cadaveric studies were also excluded. Otherwise, we did not place any restrictions on the study design due to the limited availability of data.

Teams of two reviewers participated in calibration exercises before the screening phase and subsequently screened each citation independently, then cross-verified each reference. Following the title and abstract screening portion, reviewers retrieved the full texts of eligible citations, screened each full text independently, and then cross-verified its eligibility. An additional reviewer resolved any disagreements using a modified Delphi consensus, when applicable. Reviewers then extracted data from each study independently and cross-verified the extracted data in duplicate using previously developed standardized extraction forms. Reviewers extracted the following data: study characteristics (e.g., country of origin, study design, sample size), intervention details (e.g., bolus, drip rate, aspirin dose, neuro-endovascular procedure), and treatment outcomes, including hemorrhagic and other procedural complications.

#### 2.3.4 Risk of bias assessment

Teams of two reviewers independently assessed the quality of included studies using the Newcastle Ottawa scale (NOS) and the National Institutes of Health (NIH) Quality Assessment Tool for non-randomized studies (22, 23). We used the NIH tool for case series assessment, and NOS for cohort studies. We resolved any discrepancies via consensus.

#### 2.3.5 Data synthesis and analysis

For our meta-analysis, all our assessed outcomes were categorical, which we analyzed as event rates with 95% CIs. The fixed effect model was first applied if the effect estimate was pooled from homogeneous studies; otherwise, the random effect model was used. Also, we investigated the statistical heterogeneity among studies using the I^2^ statistics chi-squared test, with *p* > 0.1 considered heterogeneous and I^2^ ≥ 50% suggestive of high heterogeneity. As our included studies for each assessed outcome were less than 10, the publication bias assessment by funnel plot and Egger’s test were not applicable (24). We conducted our meta-analysis using Comprehensive Meta-Analysis (CMA) software V3.

#### 2.3.6 Certainty of evidence assessment

We used the Grading of Recommendations Assessment, Development, and Evaluation (GRADE) to assess the strength and degree of evidence for recommendations. The certainty level in the scale consisted of four categories: high quality, which implies that additional research is not required and the confidence in the estimated effects is unlikely to change; moderate quality, which implies that further studies may impact the confidence in the estimated effects; low quality, which implies that additional research is likely to significantly impact the confidence in the estimated effects and potentially alter the estimation; and very low quality, which indicates uncertainty in the estimation.

## 3 Results

### 3.1 Retrospective study

#### 3.1.1 Baseline demographic and clinical characteristics

Seventy-six patients were included in our study. Sixty-six patients were administered protocol A, and 10 were administered protocol B cangrelor infusions. The average age was 57.2 ± 18.2 years, and 51% were males. Most were African American (65%). One quarter of our patients were active smokers (25%), and 46% had a history of hypertension.

Most of our patients underwent a neuroendovascular procedure for stroke or symptomatic carotid disease (44/76, 58%), followed by both ruptured or unruptured aneurysms (19/76, 25%). Other cases included carotid-cavernous fistulas, traumatic vessel injury or carotid blowout syndromes and idiopathic intracranial hypertension. Additional clinical and procedural characteristics are outlined in Table 2.

**Table 2:** Baseline demographic and clinical characteristics.

Characteristic	Cangrelor use (*n* = 76)
Age (mean, SD)	57.2 ± 18.2
Sex – *n* (%)	
Male	39 (51)
Female	37 (49)
Race – *n* (%)	
Black or African American	49 (66)
White	9 (12)
Other	1 (1)
Asian	2 (3)
Unknown	15 (20)
Active smoking status – *n* (%)	
Yes	19 (25)
No	38 (50)
Unknown	19 (25)
Hypertension – *n* (%)	
Yes	35 (46)
No	30 (40)
Unknown	11 (15)
Previous ischemic stroke – *n* (%)	
Yes	16 (21)
No	49 (65)
Unknown	11 (15)
Type of neurovascular pathology – *n* (%)	
Stroke/carotid disease	44 (58)
Aneurysm (ruptured or unruptured)	19 (25)
Carotid cavernous fistula	6 (8)
Traumatic vessel injury	5 (7)
Carotid blowout syndrome	1 (1)
Idiopathic intracranial hypertension	1 (1)
Location of lesion – *n* (%)	
Anterior circulation	61 (80)
Posterior circulation	13 (17)
Both	1 (1)
Unknown	1 (1)
Presence of tandem lesion^a^ – *n* (%)	
Yes	15 (30)
No	35 (70)
Baseline NIHSS score - *n* (%)	
Median (IQR)	12 (7–20)
0–10	20 (26)
11–20	20 (26)
21–30+	11 (15)
Unknown	25 (33)
	
Treatment with intravenous thrombolysis^b^ – *n* (%)	
Yes	6 (8)
No	65 (86)
Unknown	5 (7)
Type of neuroendovascular procedure – *n* (%)	
Embolization only	7 (9)
Thrombectomy only	8 (11)
Stent placement only	13 (17)
Thrombectomy and stent placement	23 (30)
Embolization and stent placement	24 (32)
Unknown	1 (1)
PRU – median (IQR)^C^	
Baseline	62.0 (19.0–152.0)
At 24–48 h	64.5 (30.8–95.5)

NIHSS, National Institutes of Health Stroke Scale; PRU, P2Y12 reaction unit; SD, standard deviation.

a Out of stroke/carotid disease patients only.

b Alteplase or tenecteplase.

c PRU values were measured in 60 patients (78%).

#### 3.1.2 Outcomes

In our cohort, we assessed for the following patient-important outcomes: mortality at 90 days, favorable functional outcome, defined by an mRS score of 0 to 2 at 90 days, and sICH. In our stroke and carotid disease group, mortality and favorable functional outcomes at 90 days were seen in 9 (21%) and 8 (24%) patients, respectively. Symptomatic intracranial hemorrhage was seen in 2 patients (7%). Two patients had major extracranial bleeding events (7%), including gastrointestinal bleeding (1/44, 3%). Most ischemic patients were discharged to an acute rehabilitation facility (18/44, 41%). In our aneurysm cohort, mortality and favorable functional outcomes at 90 days were seen in 4 (21%) and 6 (40%) patients, respectively. Among the aneurysm group, symptomatic intracranial hemorrhage was seen in 1 patient (6%), and no other bleeding events were seen. Outcomes for stroke and aneurysm groups are detailed in Table 3, and outcomes for ischemic - stroke, symptomatic carotid disease - and non-ischemic patients - aneurysms, carotid-cavernous fistulas, traumatic vessel injury, and other pathologies - are highlighted in Supplementary Table S1. Additional outcomes relating to ruptured versus unruptured aneurysms can be found in Supplementary Table S2.

**Table 3:** Clinical outcomes by pathology – stroke versus aneurysm.

Outcome	Stroke (*n* = 44)^a^	Aneurysm (*n* = 19)^b^
Safety outcomes – *n* (%)		
Mortality at discharge	5 (11)	1 (5)
Mortality at 90 days	9 (21)	4 (21)
Symptomatic intracranial hemorrhage	2 (7)	1 (6)
Asymptomatic intracranial hemorrhage	4 (13)	0 (0)
Major extracranial bleeding	2 (7)	0 (0)
Gastrointestinal bleeding	1 (3)	0 (0)
Efficacy outcomes – *n* (%)		
Favorable functional outcome (mRS 0 to 2) at discharge	15 (34)	10 (63)
Favorable functional outcome (mRS 0 to 2) at 90 days	8 (24)	6 (40)
New or recurrent strokes	4 (13)	2 (13)
In-stent thrombosis	0 (0)	1 (7)
Disposition location – *n* (%)		
Home	9 (21)	9 (64)
Subacute rehabilitation/Skilled nursing facility	5 (11)	0 (0)
Acute rehabilitation facility	18 (41)	2 (14)
Hospice/Death	8 (18)	2 (14)
Other	4 (9)	1 (7)

a Missing outcome values for 14 patients (symptomatic intracranial hemorrhage), 13 patients (asymptomatic intracranial hemorrhage), 13 patients (major extracranial bleeding), 13 patients (gastrointestinal bleeding), 11 patients (favorable functional outcome at 90 days), 14 patients (new or recurrent strokes), and 14 patients (in-stent thrombosis).

b Missing outcome values for 3 patients (symptomatic intracranial hemorrhage), 3 patients (asymptomatic intracranial hemorrhage), 3 patients (major extracranial bleeding), 3 patients (gastrointestinal bleeding), 3 patients (favorable functional outcome at discharge), 4 patients (favorable functional outcome at 90 days), 3 patients (new or recurrent strokes), and 2 patients (in-stent thrombosis).

When we compared outcomes between patients placed on different cangrelor infusion protocols, we found that mortality at 90 days occurred in 26% of patients who received Protocol A compared to 20% of patients who received Protocol B, but this difference was not significant (*p* = 0.24). Similarly, the proportion of patients with favorable functional outcomes at 90 days was also lower in the Protocol A group compared to that seen in the Protocol B group, but this difference was also not significant (40% vs. 60%, *p* = 0.31). Symptomatic intracranial hemorrhage was higher in the Protocol A group, and none was seen in the Protocol B group, but this difference was insignificant (7% vs. 0%, *p* = 0.38). Other bleeding events, such as major extracranial and gastrointestinal bleeding, were seen less in Protocol A than in Protocol B, but this difference did not meet significance (2% vs. 10%, *p* = 0.29). In-stent thrombosis occurred in 1 patient under the Protocol A group, which was attributed to nonadherence to oral antiplatelet therapy after cangrelor termination. Outcomes by cangrelor infusion protocol can be seen in Table 4.

**Table 4:** Clinical outcomes by cangrelor infusion protocol.

Outcome	Protocol A (*n* = 66)^a^	Protocol B (*n* = 10)	*p* value
Safety outcomes – *n* (%)			
Mortality at discharge	5 (8)	2 (20)	0.24
Mortality at 90 days	12 (26)	2 (20)	0.65
Symptomatic intracranial hemorrhage	4 (7)	0 (0)	N/A
Asymptomatic intracranial hemorrhage	4 (7.3)	0 (0)	N/A
Major extracranial bleeding	1 (2)	1 (10)	0.29
Gastrointestinal bleeding	1 (2)	1 (10)	0.29
Efficacy outcomes – *n* (%)			
Favorable functional outcome (mRS 0 to 2) at discharge	31 (49)	5 (50)	0.82
Favorable functional outcome (mRS 0 to 2) at 90 days	19 (40)	6 (60)	0.31
New or recurrent strokes	5 (9)	0 (0)	N/A
In-stent thrombosis	1 (2)	0 (0)	N/A
Disposition location – *n* (%)			
Home	23 (35)	5 (50)	0.34
Subacute rehabilitation/Skilled nursing facility	4 (6)	1 (10)	0.51
Acute rehabilitation facility	24 (36)	2 (20)	0.48
Hospice/Death	9 (14)	2 (20)	0.63
Other	6 (9)	N/A	N/A

a Missing outcome values for 12 patients (symptomatic intracranial hemorrhage), 11 patients (asymptomatic intracranial hemorrhage), 12 patients (major extracranial bleeding), 12 patients (gastrointestinal bleeding), 3 patients (favorable functional outcome at discharge), 19 patients (favorable functional outcome at 90 days), 17 patients (new or recurrent strokes), and 11 patients (in-stent thrombosis).

### 3.2 Systematic review

#### 3.2.1 Results of the literature search

**Figure 1:**
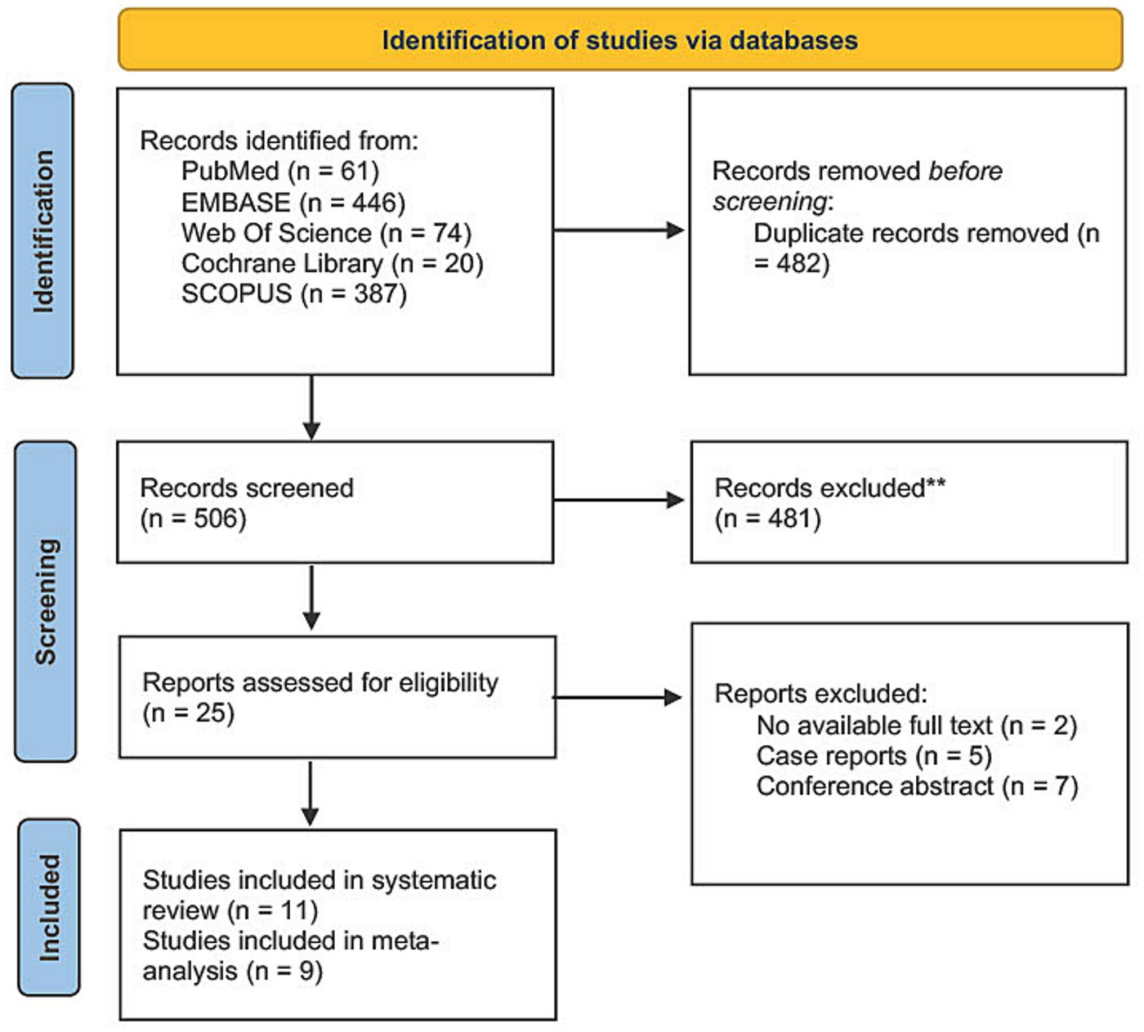
PRISMA flow diagram.

Our search method using five databases resulted in 988 studies. After duplicate elimination, 506 studies were eligible for screening. Following title and abstract screening, 25 articles were found eligible for full-text screening. Of these, 14 were excluded, leaving 11 articles that met our inclusion criteria for our systematic review (10–15, 25–29), including nine studies eligible for meta-analysis (10–12, 14, 15, 25–27, 29). Figure 1 shows the PRISMA flow diagram for the study selection.

#### 3.2.2 Characteristics of included studies

Our review included 11 studies with 298 patients, most of which were conducted in the USA. Ten studies were case series, while one study was a retrospective cohort. Five studies reported cangrelor use with stent-assisted coiling or flow-diverter stents (13–15, 25, 27), three reported using cangrelor with stent-retriever and/or aspiration (10, 12, 26), two reported using cangrelor with acute stenting (11, 29) and one reported using it with stenting or bridging (28). The bolus cangrelor regimen in included studies ranged from 5 to 40 μg/kg, while the infusion rate ranged from 0.75 to 4 μg/kg/min. In seven studies, aspirin was used as an adjuvant antiplatelet therapy with doses ranging from 75 to 500 mg daily. Supplementary Table S3 summarizes the characteristics of our included studies.

#### 3.2.3 Risk of bias assessment

Ten of our studies were assessed by the NIH tool for case series, and one study was assessed using the NOS scale for cohort studies. Nine studies showed overall good quality assessment, while two studies were of fair quality. The fair quality of the latter two studies was attributed to the inadequate description of the studied population, intervention, and statistical method used. Supplementary Tables S4 and S5 summarize the authors’ judgments using the NIH tool and NOS for each parameter.

#### 3.2.4 Outcomes

##### 3.2.4.1 Symptomatic intracranial hemorrhage

**Figure 2:**
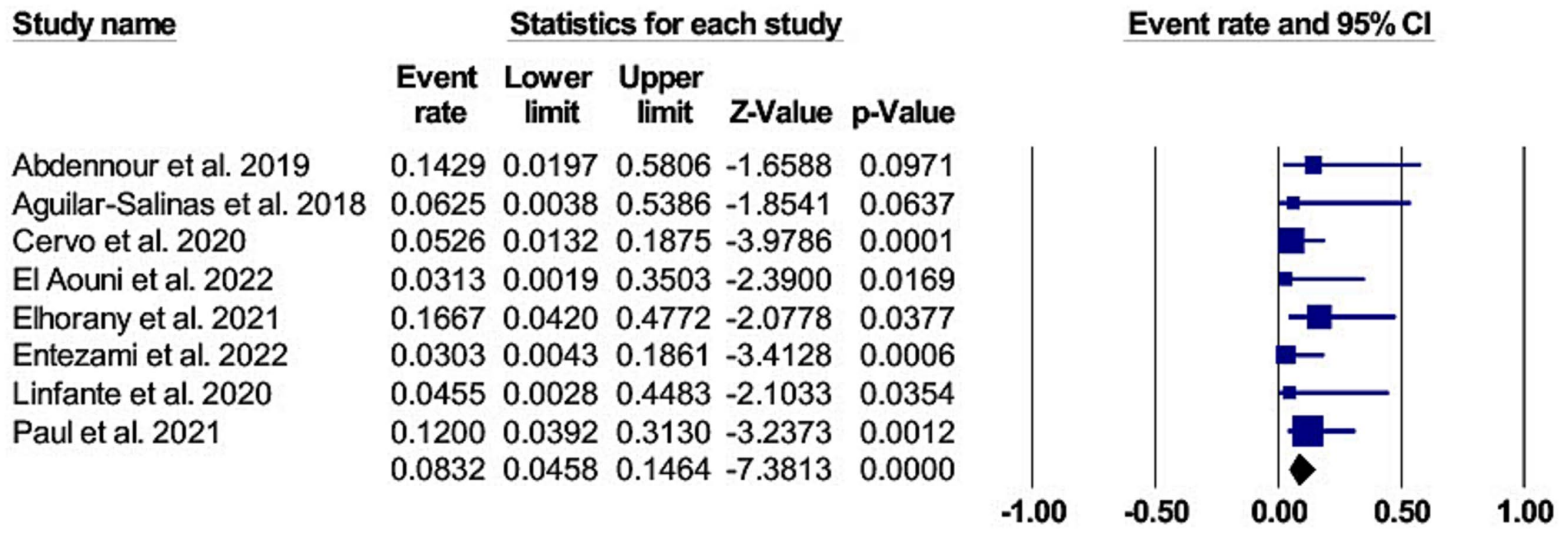
A forest plot of the asymptomatic intracranial hemorrhage.

Symptomatic intracranial hemorrhage with cangrelor use was assessed in nine studies and reported in 10 patients (7, 95% confidence interval [CI] 4.3 to 12.7). (10–12, 14, 15, 25–27, 29) The pooled studies were homogeneous, with I^2^ and *p* values were 0% and 0.90, respectively. The forest plot for sICH is shown in Figure 2.

##### 3.2.4.2 Asymptomatic intracranial hemorrhage

**Figure 3:**
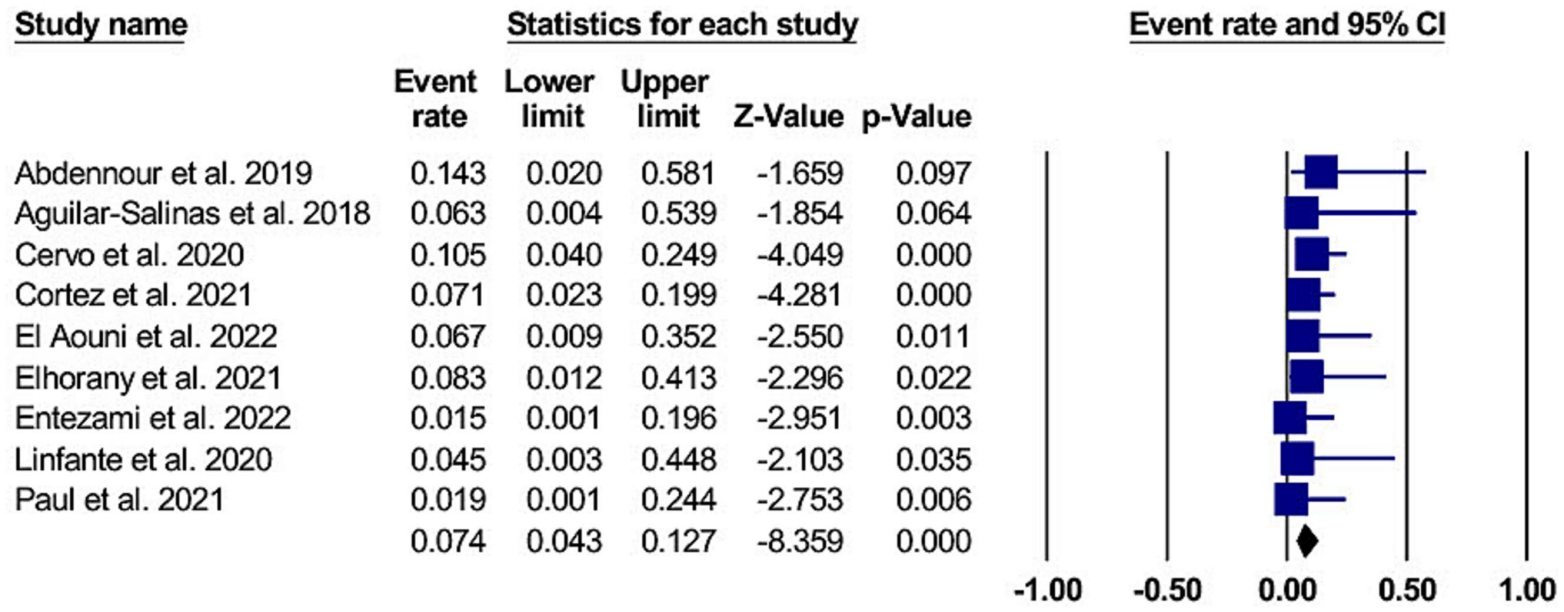
A forest plot of the symptomatic intracranial hemorrhage.

Eight studies reported the occurrence of aICH in 13 patients with cangrelor use (8, 95% CI 4.6 to 14.6) (11, 12, 14, 15, 25–27, 29). The studies were homogenous, with I^2^ and *p*-values of 0% and 0.77, respectively. The forest plot for aICH is shown in Figure 3.

##### 3.2.4.3 Retroperitoneal hematoma

**Figure 4:**
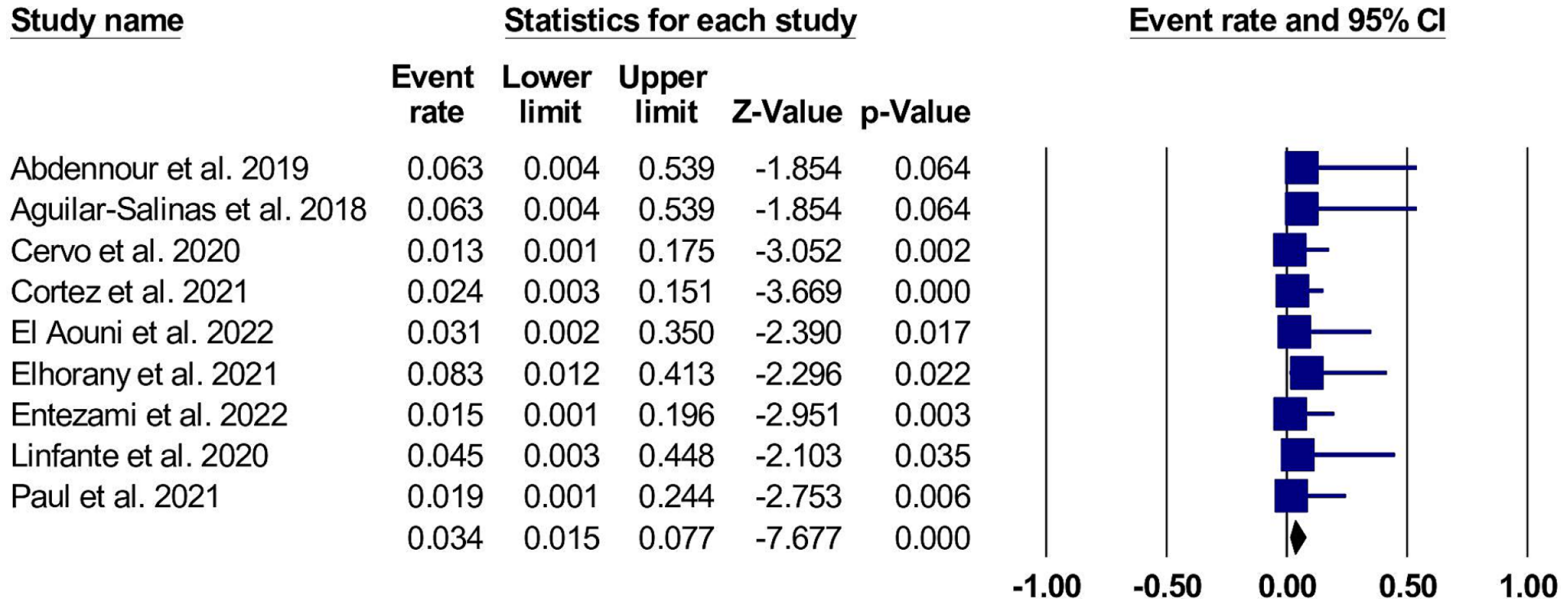
A forest plot of the retroperitoneal hematoma.

The use of cangrelor and retroperitoneal hematoma occurrence was reported in nine studies involving two patients (3, 95% CI 1.5 to 7.7). (10–12, 14, 15, 25–27, 29) The pooled studies were homogenous, with I^2^ and *p*-values of 0% and 0.97, respectively. The forest plot for retroperitoneal hematoma is shown in Figure 4.

##### 3.2.4.4 Intraprocedural thromboembolic complication

**Figure 5:**
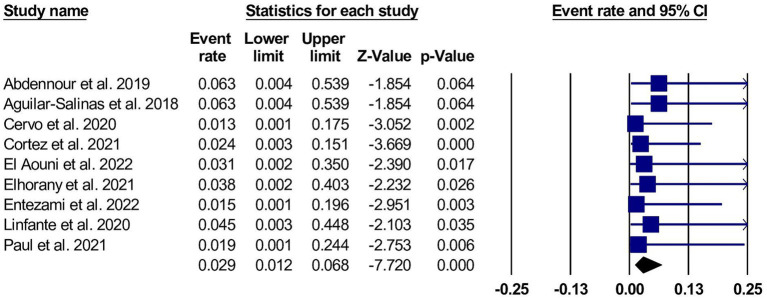
A forest plot of the intraprocedural thromboembolic complication.

Nine studies reported the occurrence of this event when cangrelor was used for neuroendovascular procedures in two patients (3, 95% CI 1.2 to 6.8). (10–12, 14, 15, 25–27, 29) The studies were homogeneous, with I^2^ and *p*-values of 0% and 0.99, respectively. The forest plot for the intraprocedural thromboembolic complication is shown in Figure 5.

#### 3.2.5 GRADE assessment

The certainty of evidence assessed in our systematic review is detailed in Supplementary Table S6. According to GRADE, all our outcomes were at a very low level of certainty, and the causes of their downgrading were: small sample size in each assessed outcome, the evidence pooled from observational studies attributed it to publication bias.

## 4 Discussion

This study is one of the first few studies to demonstrate the safety and efficacy of intravenous dose-adjusted cangrelor for acute neuroendovascular procedures and to directly compare outcomes between two different cangrelor infusion protocols in this population. Our study also uses the largest cohort of patients on dose-adjusted cangrelor from a two center, adding to the growing body of evidence that cangrelor infusion protocols require adjustment when applied to neurovascular pathologies, such as strokes and aneurysms.

Our retrospective study found that 34% of our stroke patients had a favorable functional outcome upon discharge. Among our stroke and ICA occlusion cohort, the sICH rate was 7%, with one gastrointestinal bleeding event and one femoral artery puncture with bleeding complication. When we stratified the sICH rate by cangrelor infusion protocol, we found that the sICH rate was comparable among those who received a 30 mcg/kg bolus followed by a 4 mcg/kg/min dose to those who received a 15 mcg/kg bolus followed by 2 mcg/kg/min maintenance with dose adjusted to PRU levels. Additionally, the favorable outcomes in the lower bolus group were similar to that of the higher bolus group. It is difficult to interpret these findings given the small sample size for both groups. Our updated meta-analysis demonstrates that the sICH rate with periprocedural cangrelor in a largely ischemic stroke population is approximately 7%, which is similar to results from previous meta-analyses where pooled sICH rates ranged from 6 to 9% (29, 30). On the other hand, two previous meta-analyses evaluating the use of tirofiban in stroke reported the sICH rate ranged from 7 to 9% (31, 32), and one matched cohort study of patients undergoing thrombectomy with eptifibatide reported an sICH rate of 6% (33). In a cohort study including 15 stroke patients who underwent neuroendovascular procedures and were administered abciximab, an irreversible glycoprotein IIb/IIIa inhibitor, sICH was found in 20% of patients (34). Considering these findings, cangrelor may be a reasonable alternative option. The safety of intravenous cangrelor may be attributed, in part, to its short half-life of 2 to 5 min and the restoration of platelet activity within 30 to 60 min from the time of discontinuation (35). These properties make cangrelor suitable for neuroendovascular treatment, but the dosing protocols remain under scrutiny and real-time PRU testing is not widely available. Additionally, when comparing cangrelor to other P2Y_12_ receptor inhibitors, it had several advantages that could be attributed to its nonthienopyridine structure. For example, cangrelor is a reversible P2Y_12_ receptor inhibitor and is not dependent on hepatic metabolism for activation, unlike thienopyridines such as clopidogrel and prasugrel (36–39). Moreover, cangrelor can rapidly and effectively prevent platelet aggregation, and it acts more quickly and for a shorter duration than clopidogrel (40, 41). These properties mentioned above could make it a more attractive option when emergent platelet inhibition is needed and the risk of hemorrhage is substantial.

Among our 19 aneurysm patients who underwent neuroendovascular treatment, the sICH rate was 6%. When we stratified the sICH rate according to the cangrelor infusion protocol, we found that the cardiac dosing protocol for cangrelor (Protocol B) was associated with a similar sICH rate compared to the lower aforementioned dose. In comparison to a study comparing two different dosing protocols for tirofiban, tirofiban was correlated with an increased intracranial hemorrhage rate ranging from 4 to 6% in patients with intracranial aneurysms who underwent stent-assisted coil embolization and correlated with an increased thromboembolic complication rate of 6% (42, 43). Additionally, in our meta-analysis, the thromboembolic complication was 3%, which is lower than the previously mentioned literature.

In our cohort of aneurysm patients, only one in-stent thrombosis occurred, and this was unrelated to cangrelor infusion and more related to nonadherence to oral antiplatelet therapy after discharge. A pooled analysis by Entezami and colleagues also demonstrated only one case of in-stent thrombosis in a ruptured aneurysm case due to a subtherapeutic PRU (27), which is consistent with our findings.

While our study is the first to summarize outcomes in a single cohort of patients who underwent acute neuroendovascular procedures stratified by different cangrelor infusion protocols, other studies achieved similar findings indirectly only by pooling data (27, 29). Additionally, our systematic review is the first to use the GRADE criteria to declare the certainty of our evidence. However, our study was not free of limitations. One drawback of the retrospective portion of the study was the large variation in types of procedures that were completed. Due to this it can lead to complications unique to each individual pathology, which may affect the outcomes that were observed. Additionally, the patient population is heavily skewed to one race due to the geographic location of the treating institutions. The main drawback of our meta-analysis was the study design of our included studies which were all observational studies limiting its generalizability. The small sample size of our included studies also makes the evidence imprecise and prone to type II error (false negative results). Also, because the studies were observational by design, they may contribute to publication bias even if we cannot assess this due to the small number of pooled studies per outcome (less than 10) (44, 45). For the causes mentioned above, the overall certainty of evidence for all the outcomes was very low. Therefore, larger studies using standardized treatment protocols are called upon to validate these findings.

In conclusion, our two-center study and updated meta-analysis demonstrate intravenous cangrelor’s potential safety and efficacy, highlighted by the relatively low bleeding event rates and ischemic events. Our study also calls attention to the need for higher-quality studies comparing different dosing and titration protocols of cangrelor as well as different intravenous agents in the setting of neuroendovascular procedures.

## Data availability statement

The raw data supporting the conclusions of this article will be made available by the authors, without undue reservation.

## Ethics statement

The studies involving humans were approved by The University of Chicago Institutional Review Board. The studies were conducted in accordance with the local legislation and institutional requirements. The ethics committee/institutional review board waived the requirement of written informed consent for participation from the participants or the participants' legal guardians/next of kin because of the retrospective nature of the study that did not require any direct patient contact.

## Author contributions

HD: Conceptualization, Data curation, Formal analysis, Methodology, Visualization, Writing – original draft. MA-S: Conceptualization, Data curation, Formal analysis, Writing – original draft. RM: Conceptualization, Data curation, Writing – original draft. OV: Data curation, Writing – review & editing. SK: Data curation, Writing – review & editing. ST: Data curation, Writing – review & editing. JC-P: Data curation, Writing – review & editing. AB: Data curation, Writing – review & editing. AT: Data curation, Writing – review & editing. VB: Writing – review & editing. JS: Writing – review & editing. MaH: Writing – review & editing. EC: Writing – review & editing. JB: Writing – review & editing. SM: Writing – review & editing. AM: Writing – review & editing. GD: Data curation, Writing – review & editing. MiH: Writing – review & editing. SP: Writing – review & editing. IL: Data curation, Writing – review & editing. TK-H: Conceptualization, Investigation, Methodology, Supervision, Writing – original draft.
