# Supplementary material for: Intravenous cangrelor use for neuroendovascular procedures: a two-center experience and updated systematic review
Source: Front Neurol. 2023 Dec 5;14:1304599. doi: 10.3389/fneur.2023.1304599 (PMC10728671; doi:10.3389/fneur.2023.1304599)
Supplement: Supplementary file 1 [file Data_Sheet_1.PDF]

Supplementary Table 1. Clinical outcomes by pathology – ischemic versus non-ischemic

Outcome	Ischemic (n=44)[a]	Non-Ischemic (n=32)[b]
Safety outcomes – n (%)		
Mortality at discharge	5 (11)	2 (7)
Mortality at 90 days	9 (21)	5 (21)
Symptomatic intracranial hemorrhage	2 (5)	3 (10)
Asymptomatic intracranial hemorrhage	4 (9)	1 (3)
Major extracranial bleeding	2 (5)	0 (0)
Gastrointestinal bleeding	1 (2)	1 (3)
Efficacy outcomes – n (%)		
Favorable functional outcome (mRS 0 to 2) at discharge	15 (34)	21 (72)
Favorable functional outcome (mRS 0 to 2) at 90 days	8 (24)	17 (71)
New or recurrent strokes	4 (9)	2 (7)
In-stent thrombosis	0 (0)	1 (3)
Disposition location – n (%)		
Home	9 (21)	19 (59)
Subacute rehabilitation/Skilled nursing facility	5 (11)	0 (0)
Acute rehabilitation facility	18 (41)	8 (25)
Hospice/Death	8 (18)	3 (9)
Other	4 (9)	2 (6)

[a]Missing outcome values for 14 patients (symptomatic intracranial hemorrhage), 13 patients (asymptomatic intracranial hemorrhage), 13 patients (major extracranial bleeding), 13 patients (gastrointestinal bleeding), 11 patients (favorable functional outcome at 90 days), 14 patients (new or recurrent strokes), and 14 patients (in-stent thrombosis)

[b]Missing outcome values for 3 patients (symptomatic intracranial hemorrhage), 3 patients (asymptomatic intracranial hemorrhage), 3 patients (major extracranial bleeding), 3 patients (gastrointestinal bleeding), 3 patients (favorable functional outcome at discharge), 8 patients (favorable functional outcome at 90 days), 3 patients (new or recurrent strokes), and 2 patients (in-stent thrombosis)

Supplementary Table 2. Clinical outcomes by pathology – ruptured versus unruptured aneurysm

**Outcome[a]**	**Ruptured (n=13)**	**Unruptured (n=6)**
Mortality at 90 days	5 (38.5)	0 (0.0)
Symptomatic intracranial hemorrhage	2 (15.4)	0 (0.0)
Favorable functional outcome (mRS 0 to 2) at 90 days	8 (61.5)	2 (33.3)

[a]Missing outcome values for 3 patients (symptomatic intracranial hemorrhage) and 4 patients (favorable functional outcome at 90 days)

Supplementary Table 3. Summary and characteristics of included studies

Study ID	Site	Study design	Bolus (ug/kg)	Drip Rate (ug/kg/min)	Aspirin is given, mg	Neuro-endovascular procedure	Conclusion
**Abdennour et al. 2019**	France	Case series	40	4	250	Coiling embolization and flow-diverter embolization	The symptomatic intracranial bleeding rate after IV cangrelor administration during aneurysm embolization was 4% in ruptured instances and 2.6% in unruptured cases, suggesting the procedure was safe for patients.
**Aguilar-Salinas et al. 2018**	USA	Case series	15	2	325	Acute stenting	Based on the results of this study, cangrelor is a viable alternative to conventional acute stenting for treating cerebrovascular disease.
**Cervo et al. 2020**	Italy	Case series	30	4	500	Acute stent implantation	This study's findings provided evidence that cangrelor may have a beneficial therapeutic function in controlling platelet inhibition in acute ischemic stroke operations after intracranial or extracranial stent insertion.
**Cortez et al. 2021**	USA	Case series	15-30	4-2	75–325	Acute neurovascular intervention	Cangrelor may be an option for individuals who need urgent endoluminal device intervention.
**El Aouni et al. 2022**	France	Cohort study	30	4	75	Simple coiling	This research found no statistically significant difference in safety or effectiveness between stent and flow diverter endovascular treatments for unruptured aneurysms.
**Elhorany et al. 2021**	France	Case series	30	4	250	Acute stenting	Due to its on/off action, cangrelor may be a valuable and safe antiplatelet drug in the acute stenting treatment of ischemic stroke.
**Entezami et al. 2021**	USA	Case series	30-5	0.75-1	NR	Acute stenting	Cangrelor showed promise as an alternative to standard treatments, particularly for those at high risk of bleeding.
**Entezami et al. 2022**	USA	Case series	5	0.75-1	None	Aneurysm embolization	IV cangrelor during aneurysm embolization seems safe, with symptomatic cerebral bleeding of 4% in ruptured and 2.6% in unruptured cases.
**Holden et al. 2021**	USA	Case series	5	0.75-1	NR	Neuro-endovascular stent placement	In urgent neuro-endovascular stenting and bridging cases, it was possible to use modest dosages of cangrelor with platelet function tests.
**Linfante et al. 2020**	USA	Case series	30	4	81	Stent-assisted coil embolization	IV cangrelor was a promising antiplatelet therapy option for emergency neuro-endovascular surgeries with or without oral ticagrelor.
**Paul et al. 2021**	USA	Case series	5	0.75-1	None	Acute stenting	The incidence of symptomatic cerebral bleeding in patients treated with IV cangrelor for acute ischemic stroke intervention was 6.2%.

NR: not reported.

Supplementary Table 4. Quality assessment of case series studies using NIH tool

Study ID	1. Was the study question or objective clearly stated?	2. Was the study population clearly and fully described, including a case definition?	4. Were the subjects comparable?	5. Was the intervention clearly described?	6. Were the outcome measures clearly defined, valid, reliable, and implemented consistently across all study participants?	7. Was the length of follow-up adequate?	8. Were the statistical methods well-described?	9. Were the results well-described?	Quality
**Abdennour et al. 2019**	Yes	Yes	Yes	Yes	Yes	Yes	No	Yes	Good
**Aguilar-Salinas et al. 2018**	Yes	Yes	Yes	Yes	Yes	No	Yes	Yes	Good
**Cervo et al. 2020**	Yes	Yes	Yes	Yes	Yes	Yes	Yes	Yes	Good
**Cortez et al. 2021**	Yes	Yes	No	Yes	Yes	Yes	Yes	Yes	Good
**Elhorany et al. 2021**	Yes	Yes	No	Yes	Yes	No	Yes	Yes	Good
**Entezami et al. 2021**	Yes	Yes	Yes	Yes	Yes	NR	No	Yes	Good
**Entezami et al. 2022**	Yes	Yes	Yes	Yes	Yes	NR	No	Yes	Good
**Holden et al. 2021**	Yes	Yes	Yes	Yes	Yes	NR	Yes	Yes	Good
**Linfante et al. 2020**	Yes	No	Yes	No	Yes	NR	No	Yes	Fair
**Paul et al. 2021**	Yes	No	Yes	No	Yes	Yes	No	Yes	Fair

Supplementary Table 5. Quality assessment of the cohort study using NOS tool

**Study ID**	**NOS for cohort studies**								**Quality**
	**Selection**				**Comparability**	**Outcome**			
	**Representativeness of the exposed cohort**	**Selection of the non-exposed cohort**	**Ascertainment of exposure**	**Demonstration that outcome of interest was not present at the start of the study**	**Comparability of cohorts based on the design or analysis**	**Assessment of outcome**	**Was follow-up long enough for outcomes to occur**	**Adequacy of follow-up of cohorts**	
**El Aouni et al. 2022**	*	*	*	*	*	*	*		**Good**

Supplementary Table 6. GRADE evidence profile

Certainty Assessment							Effect	Certainty	Importance
**Asymptomatic intracranial hemorrhage**									
**No. of Studies**	Designs	Risk of Bias	Inconsistency (heterogeneity in MA)	Indirectness (External Validity)	Imprecision (Small SS, Wide CI)	Publication bias	pooled proportion (95% CI)		
**8**	Case series	not serious	not serious	not serious	serious	strongly suspected	0.08 [0.05, 0.15]	very low	Important

Certainty Assessment							Effect	Certainty	Importance
**Symptomatic intracranial hemorrhage**									
**No. of Studies**	Designs	Risk of Bias	Inconsistency (heterogeneity in MA)	Indirectness (External Validity)	Imprecision (Small SS, Wide CI)	Publication bias	pooled proportion (95% CI)		
**9**	Case series	not serious	not serious	not serious	serious	strongly suspected	0.07 [0.04, 0.13]	very low	Critical

Certainty Assessment							Effect	Certainty	Importance
**Retroperitoneal hematoma**									
**No. of Studies**	Designs	Risk of Bias	Inconsistency (heterogeneity in MA)	Indirectness (External Validity)	Imprecision (Small SS, Wide CI)	Publication bias	pooled proportion (95% CI)		
**9**	Case series	not serious	not serious	not serious	serious	strongly suspected	0.03 [0.02, 0.08]	very low	Critical

Certainty Assessment	Effect	Certainty	Importance
**Intraprocedural thromboembolic complication**			

**No. of Studies**	Designs	Risk of Bias	Inconsistency (heterogeneity in MA)	Indirectness (External Validity)	Imprecision (Small SS, Wide CI)	Publication bias	pooled proportion (95% CI)		
**9**	Case series	not serious	not serious	not serious	serious	strongly suspected	0.03 [0.01, 0.07]	very low	Critical

SS: Sample Size, MA: Meta-analysis, CI: Confidence interval.
